# Unraveling Exclusive In-Plasma Initiated Oxidation Processes Occurring at Polymeric Surfaces upon O_2_ Admixtures to Medium Pressure Ar and N_2_ DBD Treatments

**DOI:** 10.3390/polym15142978

**Published:** 2023-07-08

**Authors:** Rouba Ghobeira, Parinaz Saadat Esbah Tabaei, Anton Nikiforov, Rino Morent, Nathalie De Geyter

**Affiliations:** 1 Research Unit Plasma Technology (RUPT), Department of Applied Physics, Faculty of Engineering and Architecture, Ghent University, 9000 Ghent, Belgium; parinaz.esbah@rochester.edu (P.S.E.T.); anton.nikiforov@ugent.be (A.N.); rino.morent@ugent.be (R.M.); nathalie.degeyter@ugent.be (N.D.G.); 2 Department of Chemical Engineering, School of Engineering & Applied Sciences, University of Rochester, New York, NY 14627, USA

**Keywords:** Ar/O_2_ plasma, N_2_/O_2_ plasma, in-plasma oxidation, in-situ XPS, OES, polyethylene

## Abstract

Polymeric surfaces have been increasingly plasma-activated to adopt adequate chemistries, enabling their use in different applications. An unavoidable surface oxygen insertion upon exposure to non-oxygen-containing plasmas was always observed and mainly attributed to in-plasma oxidation stemming from O_2_ impurities in plasma reactors. Therefore, this work investigates exclusive in-plasma oxidation processes occurring on polyethylene surfaces by purposely admixing different O_2_ concentrations to medium-pressure Ar and N_2_ dielectric barrier discharges (base pressure: 10^−7^ kPa). Hence, distinctive optical emission spectroscopy and in-situ X-ray photoelectron spectroscopy (XPS) data were carefully correlated. Pure N_2_ discharge triggered an unprecedented surface incorporation of large nitrogen (29%) and low oxygen (3%) amounts. A steep rise in the O-content (10%) at the expense of nitrogen (15%) was detected upon the addition of 6.2 × 10^−3^% of O_2_ to the feed gas. When the added O_2_ exceeded 1%, the N content was completely quenched. Around 8% of surface oxygen was detected in Ar plasma due to high-energy Ar metastables creating more surface radicals that reacted with O_2_ impurities. When adding only 6.2 × 10^−3^% of O_2_ to Ar, the surface O content considerably increased to 12%. Overall, in-plasma oxidation caused by O_2_ impurities can strikingly change the surface chemistry of N_2_ and Ar plasma-treated polymers.

## 1. Introduction

In the last few decades, intrinsically nonconformant polymeric surfaces have been increasingly subjected to non-thermal plasma activation to impart them with adequate chemistries, enabling their use in a wide range of technological and biomedical applications [1,2,3,4]. The high-flying position of plasma activation over other surface modification techniques is due, on the one hand, to its simplicity, versatility, solvent-free character, time efficiency, and non-invasive aspect, as its modification depth is limited to a few nanometers, thus not impeding the bulk properties of the used polymer [5,6,7]. On the other hand, the plasma capability to improve, amongst others, the surface wettability, printability, cyto- and bio-compatibility, barrier properties, and bonding characteristics of polymers has been widely demonstrated [3,8,9,10]. In general, plasma activation mechanisms lead to etching effects, cross-linking, and/or the incorporation of new functional groups onto the treated polymeric surfaces. These various reactions can actually occur simultaneously, but one can overpower the other depending on the selected experimental parameters. However, a supremacy for the grafting of chemical functionalities on non-reactive polymers is observed in the literature [7,9,11]. Nonetheless, plasma activation is limited by the multitude of non-selective functional groups that are grafted onto the surface because of the variety of chemical reactions that occur in the plasma phase. Actually, a diversified cocktail of active species that can significantly affect the surface chemistry of the polymer is present in plasma [12,13]. Despite the numerous studies aiming at fundamentally unraveling the reactions taking place on polymeric surfaces during and post-plasma activation, some ambiguities about the exact mechanisms still exist. It is anticipated that the surface modification is initiated by a series of chemical reactions generating surface radicals that can end up following different pathways depending on the used plasma gas [11,13,14]. In fact, when plasma is ignited using a noble gas such as argon or helium, the surface radicals are expected to interact with each other, causing a topology change on the polymer surface characterized by the generation of a cross-linked network. On the other hand, when plasma is sustained in a chemically reactive gas such as nitrogen or oxygen, the plasma-ensuing surface radicals are expected to react with the reactive plasma species, causing a surface functionalization theoretically distinguished by the selective incorporation of nitrogen- or oxygen-containing functional groups, respectively [5,10,15]. However, most studies involving plasma activation of polymers have revealed that these hypotheses are invalid as an unavoidable insertion of surface oxygen-containing groups on polymers exposed to plasmas sustained in non-oxygen-containing and noble gases has always been observed [7,13,14]. In fact, in the plethora of studies intending to graft nitrogen-containing functionalities on polymer surfaces exposed to N_2_ or NH_3_ plasma, X-ray photoelectron spectroscopy results have consistently shown that oxygen was more richly and easily incorporated onto the surface than nitrogen [10,15,16,17,18]. For instance, even when carefully controlling the gaseous environment to try reaching an air-free environment, Borcia et al. still grafted 10% of oxygen with a considerably lower nitrogen incorporation onto the surface of ultrahigh molecular weight polyethylene (UHMWPE) subjected to an atmospheric pressure N_2_ dielectric barrier discharge (DBD). Their observations highlighted the dominant reactivity of the trace amounts of oxygen-related species in the ambient air, as achieving a fully oxygen-free atmosphere was unsuccessful and even quasi-impossible [18]. Massines et al. have substantiated this deduction by intentionally adding only 0.2% of oxygen to a N_2_ plasma, causing a 2-factor increase in the oxygen incorporation on the surface of polypropylene at the expense of the grafted nitrogen amount, which significantly decreased [14]. As such, some researchers have concluded that even trace amounts of O_2_ can play a major role in the surface activation of polymers since it is a reactive molecule involved in a large number of oxidation reactions. Nonetheless, in contrast to the in-plasma oxidation theory, another scientific point of view states that post-plasma oxidation processes are mainly behind the significant oxygen incorporation on polymeric surfaces. In fact, when exposing the treated surface to ambient air, the formed plasma-induced carbon radicals will likely react with surrounding reactive molecules (O_2_ and H_2_O), leading to surface oxidation [10,13,19].

The unremitting debate between plasma scientists on the relative implication of in-plasma and post-plasma surface interactions in the oxidation of surfaces subjected to non-oxygen-containing discharges has been partly resolved by a few studies involving in situ XPS measurements. In this way, the implication of post-plasma oxidation can be ruled out, as the treated samples were not exposed to ambient air prior to the XPS analysis. In fact, after the treatment, the samples are placed into the XPS machine by means of a transfer vessel or a transfer arm, as in the case where the plasma reactor is directly connected to the XPS device. The majority of those studies have shed light on the fact that in-plasma oxidation processes stemming from highly reactive oxygen impurities lingering inside plasma reactors are actually largely contributing to the final surface oxygen content. When closely comparing the different studies, one could additionally conclude that the degree of in-plasma oxidation is strongly influenced by the base pressure of the plasma reactor. At considerably low base pressures in the range of 10^−6^–10^−7^ Pa, oxygen was not (or to a very low extent) incorporated on polymeric surfaces upon N_2_ or Ar plasma exposure, while at higher base pressures ranging between 10^−3^ Pa and 2 Pa, oxygen was directly grafted on Ar and N_2_ plasma-activated polymers. These conclusions were confirmed by a recent study of our research group in which comparative in situ and ex situ XPS measurements were carried out to clearly distinguish in-plasma and post-plasma mechanisms occurring on the surface of UHMWPE subjected to N_2_ and Ar DBD treatments. Oxygen contaminations could be maximally eliminated by first pumping the plasma chamber to a base pressure of 10^−7^ kPa. In situ XPS results revealed that the extent of nitrogen surface uptake that is normally significantly or even completely depleted by the presence of O_2_ in N_2_ discharges could reach an unprecedented value of more than 20% with less than 3% of surface oxygen. A slightly higher surface oxygen content (6%) was observed upon Ar plasma treatment because of the highly energetic Ar metastables that were able to generate more surface radicals able to react with O_2_ impurities in the plasma chamber. Ex situ XPS results showed trivial post-plasma oxidation, as illustrated by a slight increase in the surface oxygen content to 6% and 7% after N_2_ and Ar plasma treatments, respectively [13]. Nonetheless, despite these interesting findings showing the prevalent role of in-plasma oxidation, some important fundamental aspects have not yet been examined. All reported in situ XPS analyses were focused on discerning surface chemistries induced by discharges sustained in pure gases such as N_2_ and Ar. To the best of our knowledge, no study has investigated in-plasma-initiated oxidation processes by purposely admixing oxygen to the feed gas in order to elucidate its exclusive influence on the surface chemistry without the interference of post-plasma oxidation. By varying the concentration of the added oxygen, this strategy is believed to deliberately decorticate the exact phenomena behind unwanted or desired degrees of plasma-induced surface oxidation. As such, this paper aims at examining in-plasma-initiated oxidation processes occurring at UHMWPE surfaces when exposed to plasmas sustained in Ar and N_2_ feed gases admixed with different concentrations of O_2_ ranging from 6.2 × 10^−3^% to 5%. In contrast to the available literature mainly focused on low and atmospheric pressures, a DBD operating at a medium pressure of 5.0 kPa is used for the different treatments that are performed after evacuating the plasma chamber to a base pressure of 10^−7^ kPa. A detailed OES characterization of the different discharges, which is commonly overlooked in the literature involving in situ XPS measurements, is then carried out to unravel insights into the plasma/pure gas and plasma/gas mixture interactions. As such, the active species present in the different discharges are identified, which permits the shortlisting of possible candidates that could be involved in the surface modifications. Thereafter, an extensive in situ XPS analysis of the plasma-treated surfaces is performed, and careful correlations with the OES diagnostic results are executed. UHMWPE is selected as a model polymer given its simple chemical structure (-(CH_2_-)_n_) and chemical inertness, characterized by trivial spontaneous surface oxidation or water adsorption. As such, potential side effects on the polymer surface will be restricted, making it easier to determine and distinguish which surface phenomena are exactly taking place.

## 2. Materials and Methods

### 2.1. Materials

A commercial UHMWPE roll with a film thickness of 75 µm was purchased from Goodfellow, UK, and used without carrying out any pre-cleaning steps. Prior to the plasma treatment, the film was cut into rectangular pieces of 1.0 × 1.5 cm^2^. Pure N_2_ and Ar gases (Alphagaz 1, purity 99.999%) and different N_2_/O_2_ and Ar/O_2_ gas mixtures with 6.2 × 10^−3^%, 6.2 × 10^−2^%, 1%, 2%, 3%, 4%, and 5% of O_2_ (Alphagaz 1) were purchased from Air Liquide, Aalter, Belgium. According to the specifications provided by Air Liquide, the water and oxygen impurities in N_2_ and Ar were below 3 and 2 ppm, respectively.

### 2.2. Plasma Treatment

**Figure 1:**
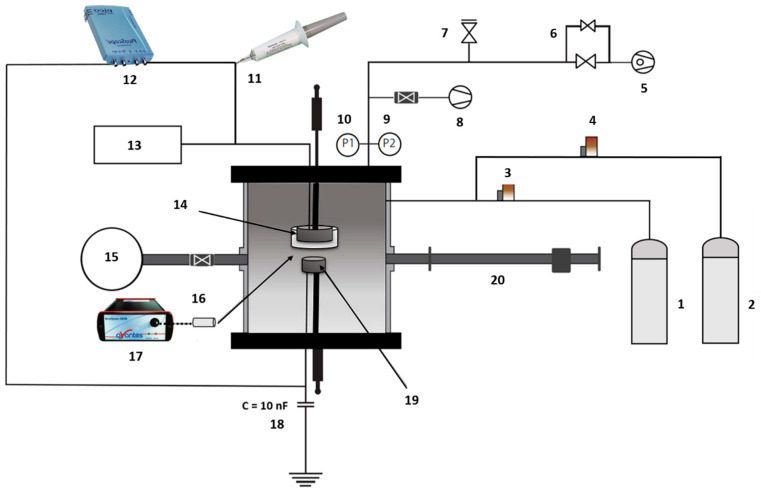
Schematic representation of the used DBD reactor: (1) N_2_ or Ar gas bottle; (2) N_2_/O_2_ or Ar/O_2_ gas mixture bottle; (3 & 4) mass flow controllers; (5) rotary vane pump; (6) needle valves; (7) vent valve; (8) turbomolecular pump; (9) Pirani gauge; (10) active inverted magnetron gauge; (11) high voltage probe; (12) oscilloscope; (13) AC power supply; (14) adjustable high power electrode; (15) XPS machine; (16) optical fiber; (17) optical emission spectrometer; (18) capacitor of 10 nF; (19) movable substrate holder (bottom electrode); (20) transfer arm.

UHMWPE samples were subjected to plasma treatment using the sub-atmospheric pressure parallel-plate DBD reactor schematized in Figure 1. The discharge was generated between two circular electrodes placed in parallel. The upper electrode, which was connected to a custom-made power supply delivering a sinusoidal high voltage at a fixed frequency of 20 kHz, is a copper plate (Ø: 48 mm) covered by an Al_2_O_3_ layer having a thickness of 3 mm and acting as a dielectric. The lower electrode, which was connected to the ground through a 10 nF capacitor, is a stainless-steel plate (Ø: 25 mm) placed at a distance of 1 mm (the gas gap) below the Al_2_O_3_ dielectric. It is worth mentioning that the electrodes were not fixed in position but could be moved up and down in the Y direction to modify the gap distance and facilitate the transfer of the sample to the XPS machine.

As already stated in the introduction, one of the main goals of this work is to elucidate the in-plasma-initiated oxidation processes by purposely admixing oxygen to the feed gas in order to elucidate its exclusive influence on the surface chemistry without the interference of post-plasma oxidation. Therefore, the DBD chamber was coupled to the XPS machine so that the treated samples could be directly moved into the XPS introduction (intro) chamber by means of a transfer arm without exposing them to ambient air.

In an effort to reduce the oxygen contamination inside the DBD to a minimal level, the plasma chamber was constantly maintained at low pressures ranging between 5 × 10^−7^ and 3 × 10^−7^ kPa by means of an Edwards turbo pump (EXT 75DX). Moreover, the polymer films were placed into the plasma chamber by first inserting them onto the lower electrode that was transferred, using a transfer arm, to the XPS intro chamber. In this way, the low pressure in the DBD chamber was not interrupted, as there was no need to open it to introduce the samples. Thereafter, the XPS intro chamber was pumped down to a pressure of 10^−3^ kPa using the rough pump assigned to the XPS machine before moving the electrode + sample into the DBD chamber. Subsequently, an additional reduction in the reactor base pressure was put into play by the simultaneous action of two turbo pumps: the one directly connected to the plasma reactor (EXT 75DX) and the one designated to evacuate the XPS intro chamber (Pfeiffer vacuum, HiPace 80). A detailed description of the procedure followed to reach the desired base pressure can be found in a previous work [13]. Once a pressure of 1 × 10^−7^ kPa was reached, a flushing step was performed by continuously sending the working gas (N_2_ or Ar) or gas mixture (N_2_ + O_2_ or Ar + O_2_) into the DBD chamber for 3 min at a constant flow rate of 3000 standard cubic centimeters per minute (sccm) as regulated by a gas mass flow controller (Bronkhorst, El-Flow^®^10 ln/min). As a result, the pressure in the DBD chamber increased to approximately 30 kPa. Finally, to carry out the actual plasma treatment, the gas flow rate was decreased to 800 sccm, the pressure in the plasma chamber was adjusted to 5.0 kPa, and the voltage power source was turned on, thus igniting plasma. In sum, the inter-electrode distance (=1 mm), total gas flow rate (800 sccm), base DBD chamber base pressure (1 × 10^−7^ kPa), working DBD pressure (5.0 kPa), and frequency of the applied voltage (20 kHz) were maintained constant, while the plasma treatment time was varied, as shown in Table 1. Moreover, for every discharge gas/gas mixture, the discharge power was fixed, and the adopted powers are also shown in Table 1. As the power was varied for every gas/gas mixture, energy density values (J/cm^2^) were calculated to enable an objective comparison between the different surface activation results. To do so, the used discharge power was multiplied by the plasma treatment time at the saturated point (corresponding to the lowest achievable WCA value, which will be explained in more detail in Section 3.3), and then the obtained value was divided by the area of the lower electrode [20].

### 2.3. Electrical and Optical Characterization

The high voltage applied to the upper electrode of the DBD reactor was measured by means of a 1000:1 high voltage probe (P6015A, Tekronix Inc., Beaverton, OR, USA), while the charge engendered on the electrodes was acquired by measuring the voltage over a 10 nF capacitor that was connected in series with the DBD reactor (Figure 1). The obtained charge-voltage waveforms, commonly known as Lissajous figures, were recorded by means of a digital oscilloscope (Picoscope 3204A, Pico Technology, Cambridgeshire, UK). Based on these Lissajous figures, the power consumed by the DBD was calculated by multiplying the electrical energy consumed per voltage cycle, which is equal to the area enclosing the Lissajous figure, by the feeding voltage frequency (20 kHz) [21].

Next to the electrical characterization of the various discharges sustained in N_2_, Ar, N_2_/O_2_, and Ar/O_2_, their optical emission spectra were also recorded to determine the excited state species present in every discharge. To do so, an optical spectrometer (S1000, Ocean Optics Inc., Dunedin, FL, USA) was used to acquire spectra in a wavelength range of 200–900 nm with a spectral resolution of 0.7 nm. In addition, a second spectrometer (AvaSpec-3648, Avantes, Apeldoorn, The Netherlands) was also employed to record spectra in a wavelength range of 300–356 nm with a resolution of 0.07 nm. The optical fiber was placed in close proximity to the front window of the DBD reactor, 20 cm away from the discharge center. The analysis of the optical emission spectroscopy (OES) results of the different discharges was carried out as described by Deng et al. [22].

### 2.4. Surface Analysis Techniques

#### 2.4.1. Water Contact Angle Measurements

To get an insight into the surface hydrophilicity of the UHMWPE films subjected to the different plasma treatments, static water contact angle (WCA) measurements were performed at room temperature. Based on these measurements, the saturation region of the plasma treatments was determined. Immediately after plasma treatment, the commercial Krüss Easy Drop system (Krüss Gmbh, Hamburg, Germany) was employed to deposit distilled water droplets (volume of 2.0 µL) on the samples. The drop profile was then instantaneously determined using Laplace-Young curve fitting. For every condition, six measurements were carried out over an extended area of a sample and averaged out.

#### 2.4.2. XPS Measurements

To assess the exclusive in-plasma-induced surface chemical composition of the UHMWPE films, XPS measurements were performed without any prior exposure of the treated samples to ambient air using a PHI Versaprobe II spectrometer connected to the DBD reactor. After a direct transfer of the treated samples to the XPS machine, their surface was excited over an area of 500 × 500 μm^2^ with an X-ray beam (size: 100 μm) generated from an Al K_α_ X-ray source (*hν* = 1486.6 eV) operating at a power of 20 W. During the measurement process, the XPS main chamber was maintained at a pressure of at least 10^−9^ kPa. The detection of the ejected photoelectrons was executed by a hemispherical analyzer placed at an angle of 45° with respect to the normal of the UHMWPE sample surface. Survey scans and individual high-resolution spectra (C1s and N1s) were acquired with pass energies of 187.85 eV (step size = 0.8 eV) and 23.50 eV (step size = 0.1 eV), respectively. All XPS data reported in this paper represent the average calculated from six measurement points that were randomly selected on each sample for every condition. The detection and quantification of the elements present on the surfaces were conducted by analyzing the survey scans using the Multipak (Version 9.6.1) software while putting into play an interacted Shirley background and the relative sensitivity factors supplied by the manufacturer of the XPS instrument. The same software was also employed to curve fit the high-resolution C1s peaks after calibrating the energy scale with respect to the hydrocarbon component (285.0 eV). To do so, Gaussian–Lorentzian peak shapes (%Gaussian > 80%) were adopted with a full width at half maximum (FWHM) of each line shape restricted below 1.5 eV.

## 3. Results and Discussion

### 3.1. Electrical Measurements

**Figure 2:**
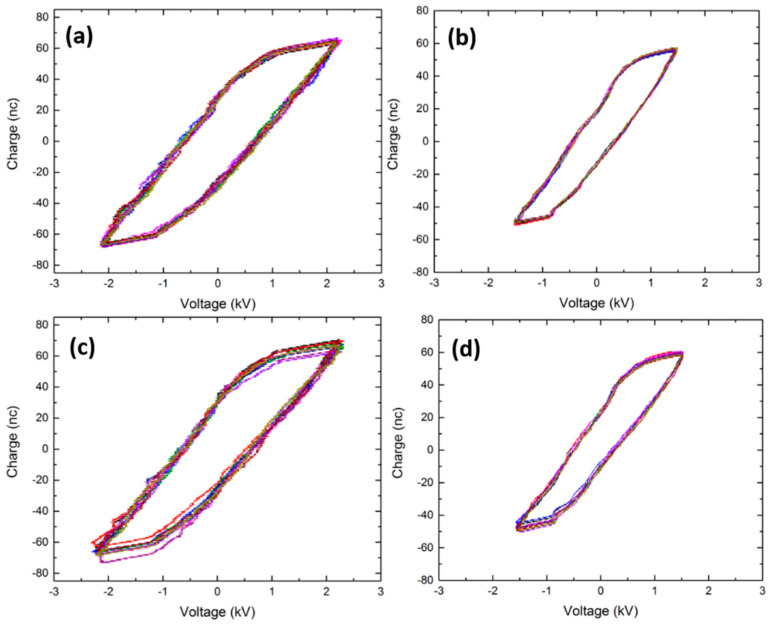
Lissajous figures of the discharges sustained in N_2_ (**a**), N_2_/O_2_ (95/5) (**b**), Ar (**c**), and Ar/O_2_ (95/5) (**d**).

In order to determine the discharge power and the mode in which the DBDs sustained in pure and mixed gases were operating, Lissajous plots were acquired. Figure 2 depicts the time-averaged Lissajous figures over 10 voltage cycles for N_2_, Ar, N_2_/O_2_ (95/5), and Ar/O_2_ (95/5) plasmas. It was noticed that by adding oxygen to the discharge, a higher power was required to provoke an electrical breakdown and, as such, ignite plasma. The reason for this observation presumably resides in the fact that the energy needed to trigger vibrational and rotational excitations of the present oxygen molecules led to disturbances of the plasma itself. As a consequence, more input power was necessary to be able to ignite plasma [23]. Moreover, given the fact that O_2_ is an electronegative gas, it can absorb electrons present in the active zone of the discharge via attachment processes through the following reaction [22]:

(1) $$O_{2}+e\;\to {O_{2}}^{-}$$

This process leads to the formation of negative ions, thus causing a reduction in the electron density in the discharge [24]. Therefore, to be able to sustain the discharge in N_2_/O_2_ and Ar/O_2_ mixtures, higher input power and electrical fields were required. In this work, the highest powers that could be applied before the formation of arcs in the plasma chamber were adopted for the pure N_2_ and Ar discharges and their counterparts containing an oxygen admixture of 5%. In order to enable a more accurate comparison between the different tested O_2_ admixtures, the same power as the one considered for the highest oxygen concentration (5%) was applied to ignite the discharges sustained in the different N_2_/O_2_ (2.4 W) and Ar/O_2_ (1.8 W) mixtures. All used powers are reported in Table 1.

When taking a look at the shape of the plotted Lissajous parallelograms, one can determine in which mode the different discharges were operating. The Lissajous figures of the pure N_2_ and the mixed N_2_/O_2_ discharges (Figure 2a,c) show both a parallelogram with rather straight but non-smooth left- and right-hand sides distinguished by very small and numerous step-like spikes. This specific shape corresponds to a filamentary discharge that is normally characterized by numerous current pulses of a nanosecond duration at every half-cycle of the applied voltage. In fact, each current pulse indicates a time-dependent spurt of charges on the electrodes and is visualized by a step on the sides of the Lissajous figures. The high number of current pulses present in a filamentary discharge explains the numerous steps of small amplitude observed on the sides of the parallelogram [25]. In contrast, the plotted parallelogram of the Ar plasma exhibits more smooth left- and right-hand sides with a barely seen evolution of one or a few depressions corresponding to wider steps (Figure 2b). Since the number of steps is equal to the number of current peaks at every half cycle of the applied voltage, one can deduce that the Ar DBD operates in a glow or multi-pulse glow discharge [25,26]. When adding oxygen to the Ar discharge, a quite similar shape of the Lissajous figure was observed but with slightly more visible steps, especially on the right-hand side of the parallelogram, thus elucidating the fact that the discharge operated in a pseudo-glow mode (Figure 2d).

### 3.2. OES Analyses of the Different Discharges

Comparative basic optical diagnostics between pure Ar and N_2_ discharges and their O_2_ admixed counterparts were carried out via OES measurements to decorticate the insights into the implicated gas/plasma interactions. As such, different excited species could be identified, thus picking out the potential candidates that were involved in the very diverse surface chemical modifications. Therefore, performing OES measurements for further correlations with in situ XPS data was of primordial relevance in this study. The spectra that are shown and analyzed in this section correspond only to plasmas sustained in pure Ar and N_2_ and in gas mixtures containing the highest oxygen concentration (5%). The other gas mixtures containing lower oxygen concentrations are not shown given the fact that the intensity of some of the peaks that appeared due to the presence of oxygen in the discharge was so low that those peaks could be barely visualized.

#### 3.2.1. N_2_ and N_2_/O_2_ Plasma Spectral Analyses

**Figure 3:**
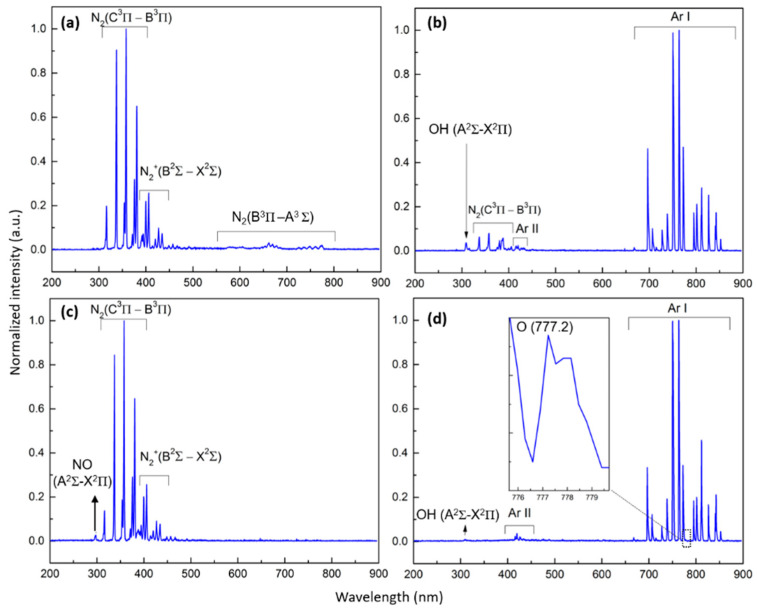
OES spectra of (**a**) N_2_ plasma; (**b**) Ar plasma; (**c**) N_2_/O_2_ (95/5) plasma; and (**d**) Ar/O_2_ (95/5) plasma.

In the case of the N_2_ plasma, the OES spectrum is dominated by intense peaks located in the wavelength range between 316 nm and 466 nm and attributed to the second positive system of N_2_, which is due to the transition C^3^Π_u_-B^3^Π_g_ [22,27]. Moreover, lower-intensity emission lines corresponding to the first negative system of the N_2_^+^ transition B^2^Ʃ_u_-X^2^Ʃ_g_ are visualized at wavelengths between 391 nm and 427 nm. Additionally, an emission of the first positive system of N_2_ based on the transition B^3^Π_g_-A^3^Ʃ_u_^+^ is perceived in the wavelength range 550 nm–800 nm and is characterized by the lowest peak intensity compared with the other bands [28] (Figure 3a). Direct electron impact excitation of relatively low energy (6.17 eV) presumably gave rise to the population of the excited N_2_ (A^3^Ʃ_u_^+^) state via the following reaction [22]:

(2) $$e+N_{2}\;(X^{1}{\Sigma_{g}}^{+})\;\to N_{2}\;(A^{3}{\Sigma_{u}}^{+})\;e.$$

Moreover, inter-vibrational collisions between N_2_ molecules can also contribute to the production of the excited N_2_ (A^3^Ʃ_u_^+^) state, as seen in Reaction (3) [22]:

(3) $$N_{2}\;(\upsilon_{1})\;+N_{2}\;(\upsilon_{2})\;\to N_{2}\;(A^{3}{\Sigma_{u}}^{+})\;+N_{2}\;(X^{1}{\Sigma_{g}}^{+}).$$

The N_2_ (C^3^Π_u_), N_2_ (B^3^Π_g_), and N_2_^+^ (B^2^Ʃ_u_) states could be generated by numerous excitations, including electron impact processes of the ground N_2_ (X^1^Ʃ_g_^+^) state and the first N_2_ (A^3^Ʃ_u_) metastable state, pooling reactions, associative excitation, and transfer of energy upon interparticle collisional processes. In fact, the ground state of N_2_ molecules present in plasma could be excited upon electron impact ionization (4) to form the N_2_ (B^3^Π_g_) state that could also be generated via the pooling Reaction (5) [29]:

(4) $$e+N_{2}\;(X^{1}{\Sigma_{g}}^{+})\;\to N_{2}\;(B^{3}\Pi_{g})\;+e$$

and

(5) $$N_{2}\;(A^{3}{\Sigma_{u}}^{+})+N_{2}\;(A^{3}{\Sigma_{u}}^{+})\;\to N_{2}\;(B^{3}\Pi_{g})\;+N_{2}\;(X^{1}{\Sigma_{g}}^{+}).$$

Moreover, given the fact that the emission peaks of the strongest intensity correspond to the transition C^3^Π_u_-B^3^Π_g,_ the excited N_2_ (C^3^Π_u_) state was also presumably spawned by direct electron impact processes (6) and collision reactions such as the polling reaction of the N_2_ (A^3^Ʃ_u_^+^) state (7) [22,29]:

(6) $$e+N_{2}\;(X^{1}{\Sigma_{g}}^{+})\;\to N_{2}\;(C^{3}\Pi_{u})\;+e$$

and

(7) $$N_{2}\;(A^{3}{\Sigma_{u}}^{+})+N_{2}\;(A^{3}{\Sigma_{u}}^{+})\;\to N_{2}\;(C^{3}\Pi_{u})+N_{2}\;(X^{1}{\Sigma_{g}}^{+}).$$

The generation of all the pre-stated excited molecular states of N_2_ is known to play an important role in the surface activation of polymers, as will be more detailed in the following XPS section [13,30].

Concerning the N_2_^+^ (B^2^Ʃ_u_^+^) state, it could be potentially formed by impact ionization of electrons via the following reaction [29,31]:

(8) $$e+N_{2}\;(X^{1}{\Sigma_{g}}^{+})\;\to {N_{2}}^{+}\;(B^{2}{\Sigma_{u}}^{+})\;+2e$$

Nonetheless, given the fact that the electron energy required to initiate Reaction (8) is rather high (>15.6 to 18.5 eV), the N_2_^+^ (B^2^Ʃ_u_^+^) excitation is more likely to occur via the collision with N_2_ (X^1^Ʃ_g_^+^) molecules having high vibrational states (9) [29]:

(9) $$N_{2}\;(X^{1}{\Sigma_{g}}^{+})\;\upsilon_{\;\geq 12}\;+{N_{2}}^{+}\;(X^{2}{\Sigma_{g}}^{+})\;\to \;{N_{2}}^{+}\;(B^{2}{\Sigma_{u}}^{+})+N_{2}\;(X^{1}{\Sigma_{g}}^{+})\;\upsilon_{\;-12}$$

It is worth mentioning that a discharge sustained at a medium working pressure of 5.0 kPa operates in a recombination mode, which means that one can neglect the diffusion loss of N_2_^+^ [32,33]. Interestingly, it was previously reported that these N_2_^+^ ions have a major impact on the effective incorporation of nitrogen-containing functionalities on an exposed polymeric surface, as will be discussed later in this paper [34]. Since no evidence for the presence of N^+^ ions is perceived in the OES spectrum, one can conclude that the N_2_^+^ state was the dominant ionic species in the DBD sustained in N_2_ at sub-atmospheric pressure. The occurrence of similar excited species following similar intensity trends was previously detected by Liu et al., who optically characterized a N_2_ DBD operating at low and medium pressures [29].

When taking a look at the OES spectrum of the N_2_/O_2_ plasma, one can distinguish two main differences compared with the spectrum of the pure N_2_ plasma: (1) the intensity of the emitted N_2_ (B^3^Π_g_-A^3^Π_u_) peaks is considerably lower, and (2) an additional emission of an NOγ peak due to the transition A^2^Ʃ^+^-X^2^Π_r_ is detected at a wavelength of 297.7 nm (Figure 3c). These two occurrences are presumably partly connected to each other given the fact that N_2_ (A^3^Ʃ_u_^+^) can act as a precursor for the formation of NO when O_2_ is present via the following reaction [35,36,37]:

N_2_ (A^3^Σ_u_^+^) + O_2_ (X^3^Σ_g_^−^) → NO (X^2^Π_r_) + NO (X^2^Π_r_). (10)

Two other frequently reported NO formation pathways are based on: (1) reactions between O atoms and N_2_ (X^1^Ʃ_g_^+^) producing NO and N atoms; and (2) reactions between N atoms and O_2_ (X^3^Ʃ_g_^−^) generating NO and O atoms [22,35,38]. Nonetheless, Gatilova et al. have demonstrated based on numerical computation that NO formation is more likely to occur via the excited metastable N_2_ (A^3^Ʃ_u_^+^) state [38]. Ono et al. have experimentally confirmed this finding by also showing that NO is mainly sourced from reactions between the N_2_ (A^3^Ʃ_u_^+^) state and exited atomic or molecular oxygen [35]. Since the OES spectrum of the N_2_/O_2_ plasma does not contain any visible peak attributed to atomic oxygen, one can conclude that the formed NO is most likely only attributed to Reaction (10) that was described in detail by Thomas et al. [36]. In fact, the absence of an atomic oxygen peak (777 nm) in the OES spectrum of N_2_ and air discharges was also reported in other studies due to the fact that O_2_ dissociation requires a relatively high electron energy above 10 eV to occur [13,22].

Once NO is formed, N_2_ (A^3^Ʃ_u_^+^) is also reported to be the principal cause of NO (A^2^Ʃ^+^) excitation responsible for the characteristic NOγ band via the Reaction (11) [37,39]:

NO (X^2^Π_r_) + N_2_ (A^3^Σ_u_^+^) → NO (A^2^Σ^+^) + N_2_ (X^1^Σ_g_^+^). (11)

Moreover, the ground state NO (X^2^Π_r_) can be electronically excited to generate NO (A^2^Ʃ^+^) levels via the following reaction [39]:

NO (X^2^Π_r_) + e → NO (A^2^Σ^+^) + e. (12)

Regardless of the formation of NO, adding O_2_ to a N_2_ discharge was revealed to quench N_2_ (A^3^Ʃ_u_^+^) leading to a decrease in its concentration. The following reaction can, for instance, occur [38]:

N_2_ (A^3^Σ_u_^+^) + O_2_ → N_2_ + O_2_. (13)

It is worth mentioning that no peaks attributed to atomic N can be detected in the OES spectra of both N_2_ and N_2_/O_2_ plasmas. Nonetheless, these N atom transitions are normally known to be very weak when compared with the transition peaks of molecular nitrogen. As such, atomic N could be potentially present but at low concentrations [34]. Such atomic N is known to be primarily produced by electron impact dissociation of excited vibrational N_2_ states via the following pathway [13,40]:

(14) $$e^{-}+N_{2}\to N_{2}(a^{1}\Pi_{g}),N_{2}(B^{3}\Pi_{g}),N_{2}(b^{1}\Pi_{u})+e^{-}\to e^{-}+N+N.$$

#### 3.2.2. Ar and Ar/O_2_ Plasma Spectral Analyses

The OES spectrum of the pure Ar plasma, presented in Figure 3b, is primarily characterized by intense atomic Ar I lines associated with the 4p → 4s transition (Racah notation) at: 696.5 nm (2p^2^→1s^5^), 706.7 nm (2p^3^→1s^5^), 727.3 nm (2p^2^→1s^4^), 738.4 nm (2p^3^→1s^4^), 750.4 nm (2p^1^→1s^2^), 763.5 nm (2p^6^→1s^5^), 772.4 nm (2p^2^→ 1s^3^), 794.8 nm (2p^4^ →1s^3^), 801.5 nm (2p^8^→1s^5^), 811.5 nm (2p^9^→1s^5^), 826.5 nm (2p^2^→1s^2^), 842.5 nm (2p^8^→1s^4^) and 852.1 (2p^4^→1s^2^) (Paschen notation) [41,42]. The 1s^2^ and 1s^4^ states correspond to the Ar resonant states, while the 1s^3^ and 1s^5^ states are accredited to the Ar metastable [43]. The dominant generation processes of these Ar excited species are the following [42]:

Ar + e → Ar(4p) + e with ΔE ≈ 13 eV, (15)

Ar + e → Ar(4s) + e with ΔE ≈ 11 eV,(16)

and

Ar(4s) +e → Ar(4p) + e with ΔE ≈ 2 eV.(17)

Moreover, Ar II lines are also observed at 415.9 nm (3p^6^→1s^5^) and 420.1 nm (3p^9^→1s^5^) but at a much lower intensity. In fact, several authors have also reported a very high emission intensity of Ar I lines compared with Ar II lines in different Ar discharges [41,44]. The generation of the observed Ar(4p) is actually a crucial step in the formation of plasma, given the fact that Ar(4p) species are a primordial station in the stepwise generation of electron–ion pairs [44]. Next to the presence of Ar emission lines, emission bands corresponding to the N_2_ second positive system due to the transition C^3^Π_u_-B^3^Π_g_ are detected at a wavelength range of 337 nm-399 nm. Moreover, the spectrum also exhibits an emission band corresponding to OH transition A^2^Ʃ^+^-X^2^Π at a wavelength of 309.9 nm. These extra N_2_ and OH optical emissions that were also previously detected in several other N_2_ discharges were attributed to the presence of impurities in plasma [13,42,45]. The OH band is most likely due to H_2_O impurities in the Ar gas, and the N_2_ band is presumably due to air traces lingering in the reactor or diffusing in the Ar gas flow. A complete evacuation of the reactor is in reality quasi-impossible, even when reaching a base pressure of 10^−7^ kPa. As such, N_2_ molecules present in the air traces could be excited to the N_2_ (C^3^Π_u_) state via electron impact, as shown in Reaction (6), or Ar(4s)/Ar(4p) as follows [42,44]:

(18) $$N_{2}\;(X^{1}{\Sigma_{g}}^{+})+\mathrm{Ar}(4s)/\mathrm{Ar}(4p)\;\to N_{2}\;(C^{3}\Pi_{u})\;+Ar.$$

The OH (A^2^Ʃ^+^) most likely originated from H_2_O molecules via the following reactions:

(19) $$H_{2}O+e\to OH\;(A^{2}\Sigma^{+})+H+e$$

and

H_2_O + Ar(4s)/Ar(4p) → OH (A^2^Σ^+^) + H + Ar. (20)

Despite the fact that the energy needed to excite the ground H state is below the energy level of the Ar(4p) state, the absence of H emission lines in the spectrum was previously attributed by Xiong et al. to the very fast quenching rate of H* by N_2_ and O_2_ that exceeds the de-excitation time [42].

When having a look at the OES spectrum of the Ar/O_2_ discharge, one can notice the disappearance of the N_2_ second positive system and a lower OH emission band intensity (309.9 nm). After careful inspection of the spectrum, one can also notice the appearance of a weak emission of O I lines (3p^5^P → 3s^5^S) at a wavelength of 777.2 nm (Figure 3d) [46]. Therefore, one can deduce that when the concentration of O_2_ in the Ar discharge reaches a certain threshold, a small amount of O atoms can be formed either by Penning ionization between Ar metastable and O_2_ (Ar* + O_2_ → 2O + Ar) or by dissociative collisions of O_2_ molecules by electrons (e + O_2_ → 2O + e) [24,47]. In contrast, the O_2_ admixture triggers a quenching of N_2_ and OH lines as a consequence of a total or partial suppression of their excitation and production mechanisms, a phenomenon that was previously observed in other studies upon adding O_2_ to an Ar discharge [23,48]. This can actually be due to the electronegativity of O_2_, which can absorb electrons via the attachment Reaction (1), resulting in a certain loss of electrons in plasma [24]. This can, as such, be partly responsible for the decrease in the OH emission line and the suppression of the N_2_ lines, as their formation through electron impact (Reactions (6) and (19)) can compete with the O_2_ attachment processes. Additionally, fewer electrons were expected to collide and excite Ar, thus reducing the production of Ar metastable. This will in turn reduce the probability of the occurrence of reactions (18) and (20) and, as such, the generation of excited N_2_ and OH bands via energy transfer from the Ar metastable. In fact, when taking a closer look at the OES spectra, one can notice that the O_2_ admixture to Ar plasma caused a decrease in the emission line at 696.5 nm and 772.5 nm corresponding to Ar metastable and an increase in the emission line at 811.5 nm due to an Ar state whose production is sensitive to low-energy electrons [49].

### 3.3. WCA Results

In order to evaluate the degree of surface hydrophilicity induced upon the exposure of UHMWPE to plasmas sustained in pure N_2_ and Ar and those admixed with 1% to 5% of O_2_, WCA measurements were performed. Within this context, it should be noted that these measurements were carried out immediately after plasma modification. Moreover, given the unfeasibility of achieving such measurements without exposing the samples to ambient air, the results were to a certain extent affected by post-plasma surface oxidation. Nonetheless, the comparison between in situ and ex situ XPS results conducted in our previous work indicated a dominance of in-plasma processes over post-plasma processes (occurring within 5 min post-treatment) in chemically modifying the surface. As such, although not totally spot-on, the WCA analysis is believed to provide a roughly accurate estimation of the optimal plasma exposure time (or energy density), leading to an exclusive in-plasma saturation of the surface chemical treatment effects. As such, the subsequent in situ XPS analysis will be limited to the obtained (sub)optimal conditions.

#### 3.3.1. N_2_ and N_2_/O_2_ Plasma Treatments

**Figure 4:**
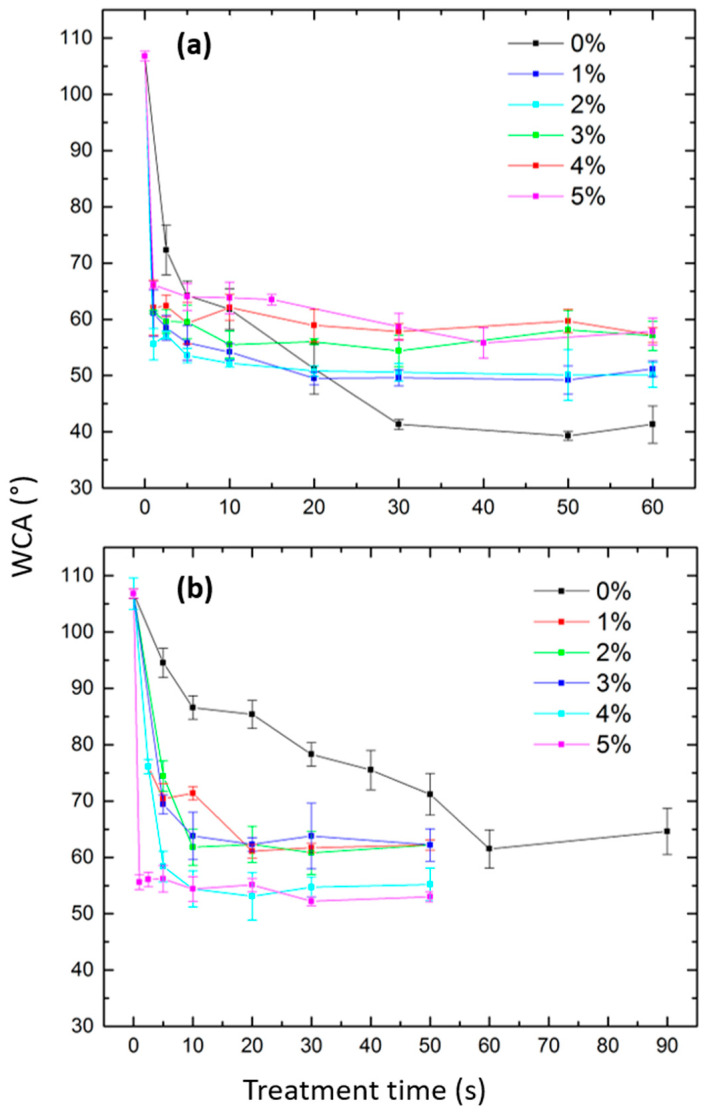
WCA evolution as a function of the treatment time upon exposure to: (**a**) N_2_ and different N_2_/O_2_ plasmas; and (**b**) Ar and different Ar/O_2_ plasmas with O_2_ admixtures ranging from 0% to 5%.

Figure 4a represents the WCA evolution of the N_2_ and N_2_/O_2_ plasma-treated UHMWPE films as a function of the plasma exposure time. In the case of the N_2_ plasma treatment, results reveal that the WCA progressively decreased from a value of 106.8° for the untreated surface to a value of 41.3° after 30 s of plasma exposure. No additional variations in the WCAs were noticed at longer treatment times, which suggests saturation of the surface modification processes. Significant differences in the WCA’s behavior were perceived when adding O_2_ to the discharge gas. Generally, the minimal attainable WCA values turned out to be notably higher than the lowest WCA value obtained upon the pure N_2_ plasma treatment. Moreover, the so-called saturated WCAs resulting from the saturation of the treatment effects were reached at shorter treatment times. When closely examining the results of the different O_2_ admixtures, one can distinguish gradual changes accompanying the increase in the O_2_ concentration from 1% to 5%. These changes are characterized by a progressive increase in the minimal reachable WCA values from 49.5° to 55.8° for O_2_ admixtures of 1% and 5%, respectively. Comparable trends in the WCA results were obtained by Massines et al., who subjected PP surfaces to N_2_ and N_2_/O_2_ plasmas [14]. As previously reported in the literature, N_2_ plasma modification normally leads to the concurrent incorporation of N- and O-containing polar groups such as amides, amines, aldehydes, alcohols, esters, and peroxides onto the surface [15,18]. Nonetheless, given the fact that oxygen was only present in the form of contamination in the pure N_2_ discharge that was characterized by the abundance of exited nitrogen species (as revealed by the OES results), one can assume that considerably more N-containing polar groups would be grafted on the N_2_ plasma-treated surface compared with the N_2_/O_2_ plasma-treated surfaces. Moreover, it also appears logical that when increasing the O_2_ concentration in the N_2_/O_2_ discharge, more O-containing functionalities would be grafted on the surface at the expense of N-containing groups. This probable decrease in the N/O ratio occurring on the UHMWPE surface when increasing the O_2_ concentration in the N_2_ discharge was presumably responsible for the observed reduced wettability. In fact, N-containing groups were reported to exhibit higher polarity compared with O-containing groups [16,50]. Moreover, the overall reduction in the saturation time upon O_2_ addition to the N_2_ discharge suggests that the incorporation of O-containing groups on the surface is faster than that of N-containing groups and might competitively oppress the latter. In order to obtain a deeper knowledge of the UHMWPE surface chemistry after N_2_ and N_2_/O_2_ plasma treatments, extensive in situ XPS analysis has been performed in the next sections.

#### 3.3.2. Ar and Ar/O_2_ Plasma Treatments

The WCA evolution of the Ar and Ar/O_2_ plasma-treated UHMWPE films as a function of the treatment time is presented in Figure 4b. An opposite trend to the one detected for the N_2_ and N_2_/O_2_ plasma-treated surfaces was perceived in this case. In fact, a progressive decrease in the WCA was also observed as the plasma treatment time increased, but the minimal attainable value for pure Ar plasma-treated surfaces (61.5°) was higher than the one reached in the case of Ar/O_2_ (95/5) plasma (52.2°). This clearly shows that the hydrophilicity of the surface increases when O_2_ is added to an Ar discharge. Moreover, the majority of plasma-induced effects took place within the first 20 s of the Ar/O_2_ plasma exposure, after which a WCA plateau was reached for all mixture ratios. However, in the case of Ar plasma modification, the saturated value was attained only after an extended treatment time of 60 s. These observations are in line with the study performed by Chen et al., in which PS surfaces were subjected to Ar and Ar/O_2_ plasmas [51]. In theory, the pure “non-reactive” Ar plasma is only expected to trigger potential changes in the surface topology without any surface functionalization. Nonetheless, given the inevitable presence of oxygen contamination in the reactor, Ar plasma was recurrently reported to exclusively graft O-containing polar groups onto treated surfaces, thus enhancing their surface wettability. The non-reactive excited species present in the discharge are able to break C-C and C-H bonds that can subsequently react with O_2_ (and OH radicals as detected in OES), thus grafting polar O-containing functionalities onto UHMWPE surfaces [6,13,52]. The very low amount of O_2_ traces present in the pure Ar discharge is likely associated with a slower surface functionalization characterized by a lower ultimate amount of grafted functionalities and hence a longer saturation time and reduced wettability. The more the discharge containsO_2_, the more O-containing polar groups can be incorporated onto its surface, leading to the detected enhanced hydrophilicity. In order to confirm these hypotheses, in situ XPS characterization has been performed on UHMWPE surfaces after exposure to Ar and Ar/O_2_ plasmas at the optimal times, giving saturated WCA values. Given the fact that the saturation times of both N_2_ and Ar plasma treatments were longer compared with the mixed plasma treatments, similar exposure times were considered for the samples treated with O_2_ admixtures of 6.2 × 10^−3^% and 6.2 × 10^−2^%, respectively. One can therefore ensure that the WCA plateau has already been attained at the chosen exposure times. The corresponding optimal energy densities that were adopted in the subsequent XPS analysis are indicated in Table 1 for N_2_, Ar, and all N_2_/O_2_ and Ar/O_2_ plasma treatments.

### 3.4. In Situ XPS Results

#### 3.4.1. N_2_ and N_2_/O_2_ Plasma Treatments

**Figure 5:**
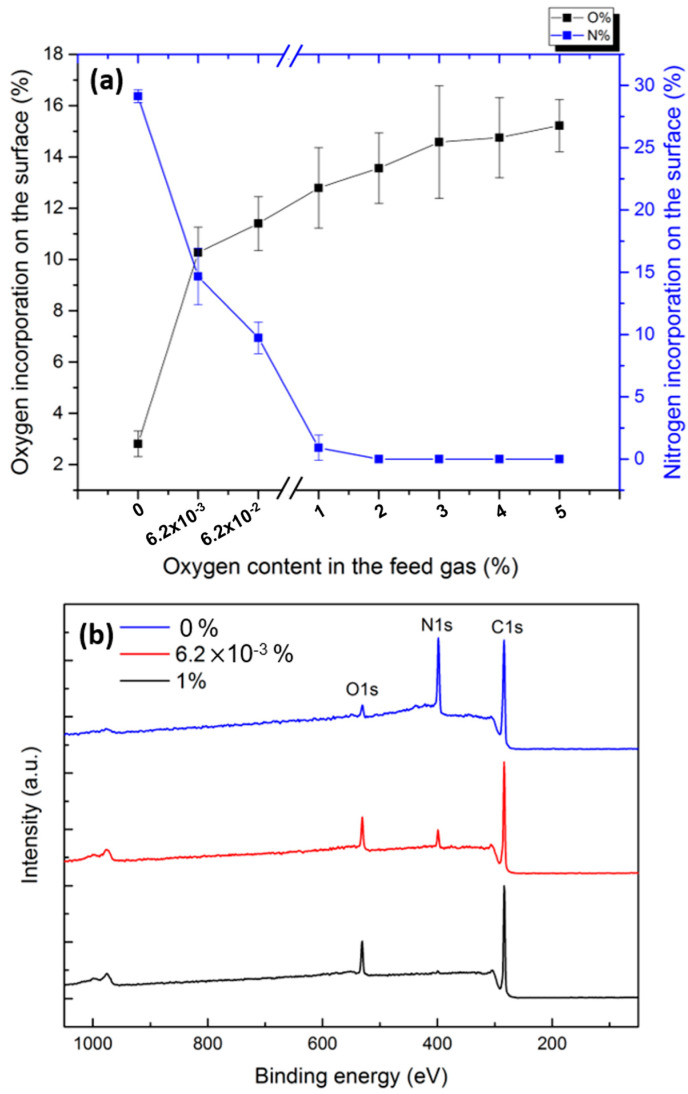
(**a**) N and O contents on the surface of UHMWPE samples subjected to plasma ignited in pure N_2_ and different N_2_/O_2_ mixtures; (**b**) Example of some of the recorded XPS survey scan spectra.

Figure 5a depicts the surface N and O contents, determined from in situ XPS survey spectra of the N_2_ and N_2_/O_2_ plasma-treated UHMWPE samples, as a function of the concentration of the added O_2_ in the feed gas. It is worth mentioning that a very small amount of surface O (≈1.1%) was already detected on the surface of the untreated UHMWPE, which could be primarily due to the inevitable surface contamination. Interestingly, plasma modification in the pure N_2_ discharge triggered the surface incorporation of an unprecedentedly large percentage of atomic N, reaching 29.1%. Two successive in-plasma mechanisms are expected to eventually lead to this abundant N incorporation. The first mechanism involves photons, electrons, and the nonreactive excited N_2_ species that were detected in the OES spectrum (N_2_ (B^3^Π_g_), N_2_ (C^3^Π_g_), and N_2_ (A^3^Ʃ_u_^+^)). Depending on their energies, all these species were previously reported to excite the polymer surface or break C-C and/or C-H bonds, thus engendering the generation of surface polymer radicals. The second mechanism implicates the reaction between these radicals and the chemically active species present in the discharge, such as atomic nitrogen states and exited N_2_^+^ ions, which eventually graft N-containing groups onto the surface [14,29,34]. As already mentioned, very weak non-visible OES transitions attributed to atomic N states, namely N(^4^S), N(^2^p), etc., known to play an important role in surface functionalization, could be potentially present [13]. Moreover, exited N_2_^+^ species, abundantly present in the discharge, were previously reported to also have a major implication in incorporating N onto polymer surfaces. Firstly, N_2_^+^ ions can acquire sufficiently high energy to break surface chemical bonds when in proximity to the substrate. Moreover, when N_2_^+^ ions recombine with the UHMWPE’s electrons, a dissociation of N_2_^+^ can occur via the following reaction:

N_2_^+^ + e_surface_ → 2N. (21)

Reaction (21) is exothermic and releases energy that is also able to break surface chemical bonds. The spawned N atoms can react with the activated sites triggered by the surface-initiated dissociative collision of N_2_^+^ ions, thus enhancing the N surface uptake [29]. Figure 5 also reveals that a small amount of oxygen was inserted onto the surface as its content increased from 1.1% to 2.8% upon N_2_ plasma treatment. This perceived slight oxygen incorporation can be attributed to the presence of oxygen traces in the used plasma reactor. This means that, despite using a base pressure of 10^−7^ kPa, not all air impurities inside the plasma chamber could be depleted. This was actually further confirmed by the appearance of excited N_2_ states in the Ar discharge (Figure 3b). Nonetheless, when compared with other studies involving N_2_ plasma treatment of UHMWPE, significantly higher incorporated oxygen amounts and, in return, lower nitrogen amounts were reported [18,53,54].

When taking a closer look at quantitative data, these differences can be clearly highlighted by N/O ratios ranging between 0.11 and 1.04 in the literature versus 10.39 in the present study [17,18,54]. This can be attributed to the presence of significantly lower concentrations of oxygen traces remaining in the DBD chamber due to the relatively low base pressure of 10^−7^ kPa. The importance of low base pressures and their primordial effect on the depletion of surface oxygen was elaborately discussed in our previous paper: the lower the oxygen contamination in the reactor is, the more nitrogen can be incorporated on the surface [13]. This shows the very high reactivity of O_2_ and its strong capacity to obviate the incorporation of surface nitrogen. The very high amount of surface nitrogen (29.1%) that was reached in this work was, to the best of our knowledge, never attained in previous studies available in the literature. Despite using a similar base pressure, a lower but still remarkably high surface nitrogen content of 20.6% was obtained in our previous work [13]. This can be attributed to the difference in discharge powers between the previous study (0.16 W) and the present study (2.1 W). In fact, by increasing the power, a more energetic plasma is ignited, thus breaking more surface bonds and triggering the formation of higher densities of excited species, such as N_2_^+^ species, which in turn enhances the surface nitrogen uptake [29,55].

The high reactivity of O_2_ in N_2_ plasmas was actually confirmed by the extreme changes in the surface elemental composition upon the deliberate addition of O_2_ to the N_2_ flow. Figure 5 reveals that when the discharge atmosphere contained only 6.2 × 10^−3^% of O_2_, about 10.2% of O was bonded to the surface of UHMWPE at the expense of the N content, which dropped to 14.6%. A progressive increase in the oxygen content to a value exceeding 14% was thereafter observed when the oxygen portion in the gas mixture was further increased from 6.2 × 10^−2^% to 5%. In return, the nitrogen incorporation onto the surface decreased significantly and became completely quenched when the added O_2_ in the feed gas exceeded 1%. Massines et al. have also revealed that by adding only 1.7 × 10^−4^% of O_2_ to N_2_ in a DBD chamber used to treat PP substrates, the surface N content decreased by a factor of 5 while the O content doubled [14]. The main difference between the OES spectra of N_2_ and N_2_/O_2_ discharges was the emission of an NOγ peak upon the addition of O_2_. This excited NO state could participate in the breakage of the C-C/C-H bonds [30]. Nonetheless, given the high stability of the ground NO state, it was most likely not implicated in the direct surface functionalization. No other excited O-containing species or atomic O could be detected in the OES results. As such, one can conclude that the high surface O content is mainly due to reactions between O_2_ molecules and plasma-induced surface radicals. Normand et al. have actually studied the respective effects of atomic and molecular oxygen on the surface functionalization of polyethylene and have come up with the following conclusions: (1) oxygen atoms act as initiators, generating surface radicals, while (2) oxygen molecules are mainly implicated in the surface functionalization upon reaction with the formed radicals [56]. In the case of the N_2_/O_2_ plasma, the initiation of the functionalization was actually driven by photons, electrons, and nonreactive excited NO and N_2_ species breaking surface bonds. Thereafter, the highly reactive O_2_ molecules, being electronegative, seemed to be attracted to the surface radicals faster than N-containing species, thus depleting their effects and ending up with a pure oxygen surface functionalization rather than a nitrogen uptake. As such, only extremely small amounts of O_2_ traces in the reactor (6.2 × 10^−3^% and 6.2 × 10^−2^%) could still allow some N-containing groups to be inserted on the surface. Figure 5b depicts the big changes in the N peak intensity of the XPS survey scan spectra recorded on UHMWPE treated with pure N_2_ plasma and N_2_ discharges admixed with 6.2 × 10^−3^% and 1% of oxygen.

**Figure 6:**
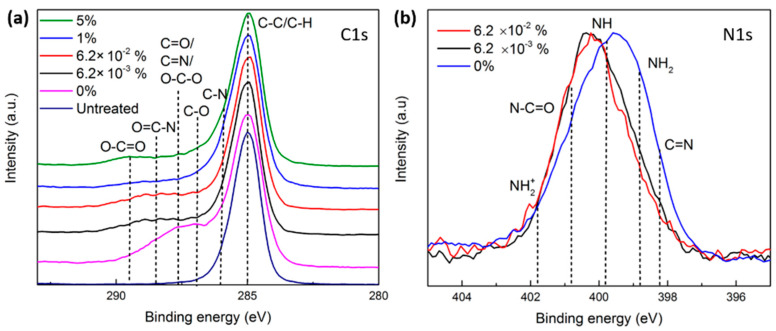
High-resolution C1s (**a**) and N1s (**b**) spectra of untreated and plasma-treated UHMWPE in pure N_2_ and different N_2_/O_2_ mixtures.

To further investigate the types of functional groups incorporated on UHMWPE surfaces upon N_2_ and N_2_/O_2_ plasma treatments, high-resolution C1s and N1s peaks were recorded and analyzed. According to literature, the C1s envelope of the untreated UHMWPE is composed of two peaks corresponding to **C**-C/C-H bonds and **C**-O bonds positioned at 285.0 eV and 286.7 eV, respectively, with the latter attributed to the detected low level of oxidation [13]. Following N_2_ and N_2_/O_2_ plasma modifications, the C1s peak exhibited additional contributions at the higher binding energy side due to the probable incorporation of different types of nitrogen- and/or oxygen-containing functionalities displaying the following extra bonds: **C-**N (285.8 eV), **C**=N/**C**=O/O-**C**-O (287.5 eV), and O**=C-**N (288.3 eV) and O**-C=**O (289.1 eV) eV [13,27,57]. Nonetheless, given the abundance of these bonds and the significant overlap between their binding energies, the C1s envelope was not fitted by separate peaks, as the resulting fittings would lead to an erroneous quantification of the different functionalities. The analysis was therefore restricted to a visual comparison between the C1s envelopes of the surfaces subjected to the different plasma treatments. For the sake of clarity, a qualitative comparison between the different N1s envelopes encompassing the following peaks was also carried out: C=**N** (398.1), **N**H_2_ (primary amines—398.8 eV), **N**H (secondary amines—399.7 eV), **N**-C=O (amides—400.8 eV), and **N**H_2_^+^ (401.8 eV) [13,58]. The N1s spectra normally exhibit a broad symmetric peak, which makes their fitting by separate peaks rather arbitrary and misguided for the extraction of meaningful data. However, it is very clear from Figure 6b that the N1s spectrum of the N_2_ plasma-treated UHMWPE is shifted towards the lower binding energy side, whereas those corresponding to the treatments with small amounts of O_2_ in the feed gas (6.2 × 10^−3^% and 6.2 × 10^−2^%) are rather shifted towards the higher binding energy side. This means that the pure N_2_ treatment incorporated mainly imine and amine groups, while the N_2_/O_2_ treatments tended to incorporate more amide groups on UHMWPE surfaces. The N1s peaks of the samples treated with N_2_/O_2_ plasmas containing 1 to 5% of O_2_ in the feed gas were not shown, as a very small amount or even no nitrogen was inserted onto the surface. The findings deduced from the N1s peaks are actually confirmed when taking a look at the C1s peaks (Figure 6a). A close inspection of the C1s envelope of the N_2_ plasma-activated substrate additionally reveals that a higher density of imine groups (C=N) compared with amine groups (C-N) is presumably inserted onto the surface. This dominance of C=N groups over C-N groups on UHMWPE surfaces subjected to N_2_ plasma is in agreement with a previous study conducted by Wagner et al. [34]. The insertion of amides upon the addition of small amounts of oxygen (6.2 × 10^−3^% and 6.2 × 10^−2^%) can be explained as follows: The non-reactive species in the discharge can trigger the generation of polymer chain radicals at α-amino carbons. Thereafter, a rapid reaction between these radicals and the O_2_ added to the feed gas takes place, thus forming peroxy radicals. Each of the two peroxy radicals can then recombine, resulting in the formation of an amide group, as explained in detail in previous studies [13,59]. When adding higher O_2_ concentrations (1–5%) to the feed gas, oxygen-containing functionalities such as C-O and O=C-O were solely incorporated onto the surface via reactions between O_2_ and plasma-induced surface radicals, similar to the reactions occurring post-plasma treatment and described in detail in our previous study [13].

Overall, the surface reactions occurring upon the exposure of UHMWPE to N_2_ and N_2_/O_2_ plasmas can be categorized into three different pathways:

- In pure N_2_ plasma, reactions of carbon-centered surface radicals (C^•^) with active species (N atoms and N_2_^+^ ions) resulted in the formation of N-containing functional groups, namely C-N and C=N, on the surface.
- In N_2_/O_2_ plasmas containing very low amounts of O_2_ (6.2 × 10^−3^% and 6.2 × 10^−2^%), the formed polymer radicals reacted with reactive nitrogen species and O_2_ in the feed gas, inserting N- and O-containing functional groups such as C-O, C=N, and O=C-N onto the surface.
- In N_2_/O_2_ plasmas sustained in gas mixtures containing O_2_ concentrations exceeding 1%, the plasma-induced surface radicals solely reacted with O_2_ molecules that completely depleted the incorporation of N onto the surface. In fact, O_2_ molecules are highly reactive and electronegative, thus rapidly reacting with the surface radicals and overpowering the reactions with nitrogen species.

#### 3.4.2. Ar and Ar/O_2_ Plasma Treatments

**Figure 7:**
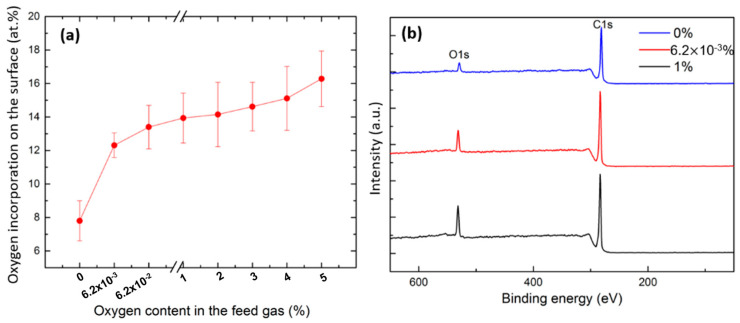
(**a**) O content on the surface of UHMWPE samples subjected to plasma ignited in pure N_2_ and different N_2_/O_2_ mixtures; (**b**) Example of some of the recorded XPS survey scan spectra.

Figure 7 depicts the surface O content of the samples modified with Ar and Ar/O_2_ plasmas as a function of the concentration of the added O_2_ in the feed gas, as determined from in situ XPS survey scans. Upon plasma modification in the pure Ar discharge, the surface oxygen concentration remarkably increased from 1.1% (pristine sample) to 7.8%. In theory, a pure Ar atmosphere should not contain chemically reactive species leading to oxygen incorporation onto the surface. However, this notable amount of inserted surface oxygen could be to a large extent attributed to the presence of residual air lingering in the plasma chamber in addition to impurities present in the Ar feed gas and potential outgassing of the electrodes [13]. The incomplete evacuation of the plasma chamber was actually confirmed by the emission of excited N_2_ lines in the OES spectrum (Figure 3b). As such, given the fact that the base pressure pre-plasma treatment was set to 10^−7^ kPa, one can conclude that the presence of oxygen traces is sufficient to induce oxygen-based surface functionalization. This finding is actually in agreement with previous literature studies showing that the effect of even very small concentrations of impurities in the feed gas (<3 ppm) is associated with pronounced variations in the surface chemical composition upon plasma modification [13,14,15,60]. Nonetheless, it is worth emphasizing on the fact that in this paper, the O/C (0.08) ratio of Ar plasma-treated UHMWPE iwass substantially lower than the ones obtained in other studies in which higher plasma base pressures were adopted. For instance, Teodoru et al. and Aziz et al. have subjected UHMWPE to Ar DBD treatments where higher base pressures were used and have obtained a surface O/C ratios of 0.17 and 0.22, respectively [17,61]. When comparing between the surface oxygen incorporation occurring in pure Ar plasma (O content: 2.8%) and pure N_2_ plasma (O content: 7.8%), one can clearly notice its more prominent incidence in Ar plasma. This can be presumably attributed to the more energetic Ar metastables generated in Ar plasma compared with the excited species produced in N_2_ plasma. These metastables that were detected in the OES together with some excited N_2_ species, can very efficiently break surface C-C and C-H bonds thus creating more surface radicals that can react with O_2_ [62]. As already mentioned, oxygen molecules are highly reactive which leads to a rapid initiation of reactions between them and the plasma-induced radicals thus functionalizing the surface [56]. Moreover, Ar metastables could as well efficiently dissociate the H_2_O molecules present in the feed gas and plasma chamber thus producing OH radicals as was seen in the OES spectrum. These radicals are known to play a major role in the chemical processes leading to the incorporation of O-containing groups onto UHMWPE; hence the higher surface O content [13].

The moment 6.2 × 10^−3^% of oxygen was added to the gas flow, a sharp increase in the surface oxygen content was detected (12.3%). Moreover, by adding more oxygen to the feed gas (from 6.2 × 10^−3^ to 5%), a further progressive and less steep increase in the O content reached a value of 16.3%. This shows that the evolution of the incorporated oxygen amount onto the surface runs in parallel with the amount of added O_2_ in the gas mixture. This evolution is nicely represented by the decrease in the O peak intensity in the XPS survey scan spectra (Figure 7b). These results are in line with a previous study performed by Gerenser et al. [19]. In fact, when more O_2_ is present in the discharge, more surface radicals are allowed to react with O_2_ molecules instead of interacting with each other, which eventually leads to more O-containing functionalities inserted on the surface with a lower degree of cross-linking. When taking a look at the OES spectrum of the Ar/O_2_ discharge, one can detect the disappearance of the excited N_2_ emission lines, which is compensated by the appearance of a small atomic O line (Figure 3d). This atomic O was reported to be very powerful in initiating surface functionalization by generating surface radicals [56].

**Figure 8:**
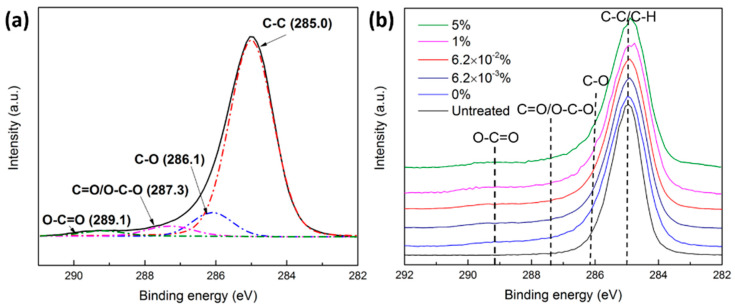
(**a**) Representation of a characteristic C1s curve fitting in case of Ar and Ar/O_2_ plasma treatments; (**b**) High-resolution C1s spectra of untreated and plasma-treated UHMWPE in pure Ar and different Ar/O_2_ mixtures.

In order to get more insight into the types and relative concentrations of the different oxygen-containing functionalities inserted on the surface of UHMWPE upon Ar and Ar/O_2_ plasma treatment, high-resolution C1s peaks were recorded and analyzed. Such C1s envelopes induced by Ar and Ar/O_2_ plasma treatments can be decomposed into four distinctive bonds, as shown in Figure 7a: **C-**C, **C-**O, **C=**O/O-**C**-O, and O**-C=**O at 285.0, 286.1, 287.3, and 289.1 eV, respectively [6,7,13,20]. Given the fact that no nitrogen-containing functionalities were incorporated onto the surface, the C1s envelope is fitted by the different peaks attributed to the abovementioned bonds (Figure 8). Since these peaks are relatively distant from each other, a fairly accurate quantification of the different functional groups can be obtained.

Based on the carried-out C1s XPS fittings, the relative concentration of each carbon-containing group has been acquired, and the results are presented in Table 2. Results reveal that Ar and Ar/O_2_ plasma treatments simultaneously introduced different oxygen-containing functional groups, such as C-O, C=O/O-C-O, and O-C=O bonds. However, most oxygen was incorporated in the form of C-O bonds (≈10%) on all Ar and Ar/O_2_ plasma-modified UHMWPE samples, which is consistent with other ex situ XPS results available in the literature [17,63]. It is worth mentioning that the relative concentration of all added groups was more or less similar as the oxygen concentration increased in the feed gas. As such, one can conclude that by adding more oxygen to the feed gas, the overall surface O content increased, but this oxygen was incorporated in the form of relatively equal portions of chemical bonds in the following order of descending concentrations: C-O (≈10%), C=O/O-C-O (≈3.5%), and O-C=O (≈2%). These groups were inserted on the surface by different chemical processes triggered by the reaction of O_2_ with the plasma-induced carbon-centered surface radicals, as described in our previous paper [13].

## 4. Conclusions

In the present work, an extensive comparative study on in-plasma processes occurring at UHMWPE surfaces subjected to plasmas sustained in pure Ar and N_2_ versus different Ar/O_2_ and N_2_/O_2_ mixtures (O_2_ concentration ranging from 6.2 × 10^−3^% to 5%) was conducted. As such, exclusive in-plasma-initiated oxidation processes and their effects on the final surface chemical composition were examined without the interference of post-plasma oxidation. To do so, OES measurements were first carried out to determine the influence of the different gas compositions on the generation of excited species, which played a major role in the subsequent surface chemical modifications. Results revealed the predominant presence of different excited molecular nitrogen species (N_2_ (C^3^Π_u_), N_2_ (B^3^Π_g_), and N_2_ (A^3^Ʃ_u_^+^)) and ionic nitrogen states (N_2_^+^ (B^2^Ʃ_u_) in the pure N_2_ discharge. When O_2_ was added to N_2_, an additional OES emission line attributed to excited NOγ was detected. The pure Ar discharge was primarily characterized by Ar resonant states and metastables in addition to excited OH (A^2^Ʃ^+^) and N_2_ (C^3^Π_u_-B^3^Π_g_). The latter two excited species were attributed to the presence of air and H_2_O traces in the plasma reactor. The moment O_2_ was added to the Ar flow, a weak emission of an atomic O line and the disappearance of the excited N_2_ species were noticed. Thereafter, profound correlations between OES results and in situ XPS measurements were carefully conducted to shortlist the species interacting with the surface and leading to the obtained diverse surface chemistries. Interestingly, plasma modification in the pure N_2_ discharge triggered the surface incorporation of an unprecedentedly large percentage of atomic N, reaching 29.1%. In fact, photons, electrons, and excited N_2_ molecules present in the discharge were able to generate surface polymer radicals that in turn reacted with the chemically active species, namely atomic nitrogen, nitrogen metastables, and the exited N_2_^+^ species, thus grafting N-containing groups onto the surface. The very low reactor base pressure of 10^−7^ kPa was actually behind the very low surface oxygen incorporation (2.8%). However, more surface oxygen was detected after Ar activation, which was likely due to the high-energy Ar metastables more efficiently breaking surface C-C and C-H bonds thus creating more surface radicals that could react with the highly reactive O_2_ impurities. Moreover, Ar metastables could as well efficiently dissociate H_2_O molecules present in the feed gas thus producing OH radicals known to play a major role in the incorporation of O-containing groups onto surfaces. A very high reactivity of O_2_ in N_2_ plasmas was perceived via the detected extreme changes in the surface elemental composition upon the deliberate addition of O_2_ to the N_2_ flow. In fact, a steep rise in the O surface content at the expense of the N content (14.6%) was detected upon the addition of only 6.2 × 10^−3^% of O_2_ to the feed gas. When the added O_2_ exceeded 1%, the nitrogen incorporation onto the surface became completely quenched. In this case, the surface radicals created by the non-reactive plasma species were likely rapidly reacting with the highly reactive and electronegative O_2_ molecules in a way that overpowered the reactions with nitrogen species. The moment a small concentration of O_2_ was added to the Ar gas flow, a sharp increase in the surface oxygen content was also detected. Thereafter, the evolution of the surface oxygen amount ran in parallel with the amount of extra O_2_ added to the gas mixture. In fact, when more O_2_ was present in the discharge, more surface radicals were allowed to react with O_2_ molecules instead of interacting with each other, which eventually led to more O-containing functionalities being inserted on the surface. Overall, one can conclude that in-plasma oxidation processes initiated by the presence of O_2_ in the plasma reactor can, depending on the O_2_ concentration, strikingly change the surface chemistry of UHMWPE exposed to N_2_ and Ar plasmas.

## Figures and Tables

**Table 1:** Overview of operational parameters adopted during the different plasma treatments sustained in N_2_, Ar, and the different N_2_/O_2_ and Ar/O_2_ mixtures.

Plasma Gas	Discharge Power (w)	Treatment Time (s)	Energy Density at Saturated Point (J/cm^2^)
N_2_	2.1	0–60	12.8
N_2_/O_2_ (O_2_: 6.2 × 10^−3^%)	2.4	30	14.6
N_2_/O_2_ (O_2_: 6.2 × 10^−2^%)	2.4	30	14.6
N_2_/O_2_ (99/1)	2.4	0–60	9.7
N_2_/O_2_ (98/2)	2.4	0–60	9.7
N_2_/O_2_ (97/3)	2.4	0–60	14.6
N_2_/O_2_ (96/4)	2.4	0–60	14.6
N_2_/O_2_ (95/5)	2.4	0–60	19.5
Ar	1.2	0–90	14.6
Ar/O_2_ (O_2_: 6.2 × 10^−3^%)	1.8	60	22.0
Ar/O_2_ (O_2_: 6.2 × 10^−2^%)	1.8	60	22.0
Ar/O_2_ (99/1)	1.8	0–50	7.3
Ar/O_2_ (98/2)	1.8	0–50	3.6
Ar/O_2_ (97/3)	1.8	0–50	7.3
Ar/O_2_ (96/4)	1.8	0–50	7.3
Ar/O_2_ (95/5)	1.8	0–50	14.6

**Table 2:** Relative concentration (in %) of carbon containing functionalities on UHMWPE films after Ar and Ar/O_2_ plasma treatments.

	Untreated	Pure Ar	Ar + O_2_ (6.2 × 10^−3^%)	Ar + O_2_ (6.2 × 10^−2^%)	Ar + O_2_ (1%)	Ar + O_2_ (2%)	Ar + O_2_ (3%)	Ar + O_2_ (4%)	Ar + O_2_ (5%)
**C**-C	95.3 ± 0.5	83.8 ± 1.3	84.3 ± 0.5	84.4 ± 1.1	82.7 ± 1.8	85.1 ± 1.7	83.6 ± 0.8	83.3 ± 1.1	82.1 ± 1.7
**C**-O	4.6 ± 0.5	10.7 ± 0.9	10.1 ± 0.3	9.8± 0.6	10.9 ± 1.6	9.4 ± 0.9	10.9 ± 1.0	10.9 ± 1.3	10.8 ± 1.5
**C**=O**/**O-**C**-O	-	3.6 ±0.1	3.3 ± 0.3	3.3 ± 0.2	3.8 ± 0.4	3.2 ± 0.3	3.9 ± 0.4	3.5 ± 0.3	4.3 ± 0.5
O-**C**=O	-	1.6 ± 0.3	2.1 ± 0.2	2.2 ± 0.3	2.3 ± 0.5	2.1 ± 0.5	2.1 ± 0.3	2.1 ± 0.4	2.7 ± 0.4

## Data Availability

Data is contained within the article.
